# Comparison of Scheimpflug tomography, Placido disc, and combined Placido Scheimpflug in the measurement of pupil offset in myopic population

**DOI:** 10.3389/fmed.2024.1490674

**Published:** 2024-11-05

**Authors:** Jiliang Ning, Lijun Zhang

**Affiliations:** ^1^Department of Ophthalmology, The Third People’s Hospital of Dalian, Dalian, China; ^2^Department of Ophthalmology, Dalian Municipal Eye Hospital, Dalian, China; ^3^Liaoning Provincial Key Laboratory of Cornea and Ocular Surface Diseases, Dalian, China; ^4^Liaoning Provincial Optometry Technology Engineering Research Center, Dalian, China

**Keywords:** pupil offset, kappa angle, Sirius, Pentacam, Keratron Scout, Scheimpflug tomography, Placido disc

## Abstract

**Introduction:**

This study aimed to compare the consistency of pupil offset measurements obtained using the Pentacam, Keratron Scout, and Sirius devices.

**Methods:**

This retrospective cross-sectional study included 146 young myopic individuals (292 eyes) scheduled for refractive surgery at Dalian Third People’s Hospital between January 2023 and December 2023. Three devices were utilized to measure the chord mu of the pupil deviation along with the Cartesian distances of the X and Y coordinates (Px, Py) associated with the pupil offset. Repeated-measures analysis of variance was used to compare differences in pupil offset acquisition across various devices. Additionally, the intraclass correlation coefficient (ICC) and Bland–Altman plot were utilized to assess the consistency among the three devices.

**Results:**

Chord mu, measured using the Pentacam, Keratron Scout, and Sirius devices, were 0.18 ± 0.10, 0.21 ± 0.11, and 0.18 ± 0.11, respectively. The Px values were 0.00 ± 0.14, -0.02 ± 0.16, and -0.01 ± 0.13, respectively, while the Py values were 0.09 ± 0.13, 0.10 ± 0.15, and 0.10 ± 0.13. The ICCs for the three device measurements, chord mu, Px, and Py, were 0.817, 0.900, and 0.855, respectively. When comparing the three devices, the 95% limits of agreement (LoA) for mu and Px measured using the Sirius and Keratron Scout were the narrowest, ranging from −0.15 to 0.08 and −0.11 to 0.13, respectively. Additionally, the 95% LoA for Py measured using the Sirius and Pentacam was the narrowest, ranging from −0.13 to 0.15. The pupil centers in both eyes were predominantly located above the apex of the cornea.

**Conclusion:**

Sirius, Keratron Scout, and Pentacam have good consistency in pupil shift measurement in young myopic patients, and the three devices can be used as references in clinical practice.

## 1 Introduction

The distance and direction of the pupil center relative to the corneal apex, measured in the corneal plane, are referred to as the pupillary offset (1). The corneal apex is the point where the coaxial visual light is reflected, corresponding to the first Purkinje image. Since it remains unaffected by variations in pupil size, the corneal apex serves as a more stable reference point than the pupil center, positioning it closer to the visual axis corneal intercept (2). In corneal refractive surgery, precise positioning of the cutting center is essential for achieving optimal postoperative visual outcomes. Eccentricity during the cutting process can increase higher-order corneal aberrations, leading to visual quality issues, including glare, halos, and monocular diplopia (3–5). Research indicates that compared to the pupil ablation center, the corneal vertex center alignment strategy yields superior visual and refractive outcomes (6). Consequently, the accurate measurement of pupillary shift prior to refractive surgery is essential for enhancing postoperative visual quality. The Pentacam (Oculus Inc., Wetzlar, Germany) acquires corneal tomography images using a high-resolution rotating Scheimpflug camera, while the Keratron Scout (Optikon, Rome, Italy) captures the tangential curvature topography of the cornea through a Placido disk. Additionally, the Sirius (Schwind Eye-Tech-Solutions Ltd., Germany) system gathers corneal data by integrating both a Scheimpflug camera and a Placido disk. These devices are widely used to obtain pupil offset data (7, 8). The purpose of this study was to investigate the consistency of Pentacam, Keratron Scout, and Sirius in measuring pupil offset in myopia refractive surgery candidates and to provide a reference for refractive surgery ablation centers.

## 2 Materials and methods

### 2.1 Research subjects

This retrospective cross-sectional study involved 146 myopic individuals (292 eyes) who were scheduled to undergo refractive surgery at Dalian Third People’s Hospital between January 1, 2023, and December 31, 2023. The cohort consisted of 81 males and 65 females, with ages ranging from 18 to 52 years. Inclusion criteria included stable refraction over the past 2 years, cessation of soft contact lens use for at least 1 week, rigid gas permeable (RGP) lens use for a minimum of 1 month, and orthokeratology lens use for at least 3 months. Additionally, participants needed to have a best-corrected visual acuity of ≥0.8 (on the decimal chart). The exclusion criteria were congenital eye developmental abnormalities, glaucoma, cataracts, keratoconus, frustrated keratoconus, active corneal inflammation, severe dry eye, corneal scarring, and a history of eye trauma or surgery. Additionally, individuals who were unable to cooperate during the examination were excluded. This study adhered to the Declaration of Helsinki and was approved by the Ethics Committee of the Dalian Third People’s Hospital (No. 2024–101-001). In accordance with the requirements set forth by the ethics committee, the waiver of patient informed consent was granted. The authors did not have access to any information that could identify individual participants during or after the data collection process.

### 2.2 Method of examination

All measurements in this study were conducted in a uniform, windowless examination room, utilizing indoor light as the sole lighting source (ambient brightness set at 60 lux). This approach was implemented to minimize the influence of external light sources during the examination and to ensure a consistent environment for the assessment of all three devices when examining patients. All measurements were performed by the same examiner. The subject was instructed to maintain an upright head position with the lower jaw resting on the chin rest and the forehead positioned close to the forehead rest position. Prior to measurement, the subject was asked to blink several times to ensure tear film stability. The subjects were then required to keep their eyes open and focus on the target, with three consecutive measurements taken for each eye. The best quality image (quality specification = OK) was used for analysis to ensure the accuracy and reliability of the results. When measuring subjects, it is essential to maintain an interval of more than 5 min between the use of different devices. Sirius and Keratron Scout utilize a polar coordinate system to represent the pupil offset, that is, the planar distance (chord mu) and the angle between the pupil center and the corneal apex (the coordinate origin). The Pentacam measurement results utilize the XY Cartesian coordinate system, in which the pupil offset is defined as the vertical distance (Px and Py) between the corneal vertex, which serves as the coordinate origin, and the center of the pupil. The plane distance was calculated in millimetres (mm). The two coordinate systems were converted using built-in formulas in Excel software (2019, Microsoft Corp., Redmond, USA).

### 2.3 Statistical analysis

Statistical analyses and graph creation were conducted using MedCalc software (version 22.001; MedCalc, Ostend, Belgium) and OriginPro (version 2024; OriginLab, Northampton, USA). Measurement data are presented as mean ± standard deviation, range, and 95% confidence interval. The Shapiro–Wilk test was used to assess data normality. Repeated-measures analysis of variance was used to compare the overall differences in the chord my and Px and Py components. Assuming a significance level (α) of 0.05 for a two-sided test and a type II error (β) of 0.1, which corresponds to a test power of 0.9, the calculated sample size is determined to be at least 50 participants. The Bonferroni test was used for *post hoc* pairwise comparisons. The intraclass correlation coefficient (ICC) was used to assess the reliability of the measurements obtained using the three devices. Following the guidelines established by Terry K. Koo, an ICC estimate with a 95% confidence interval of <0.5 signifies poor reliability, while a range between 0.5 and 0.75 indicates acceptable reliability. Reliability in the medium range, classified as values between 0.75 and 0.9, indicates good reliability, while values above 0.90 signify excellent reliability (9). Bland–Altman analysis was employed to assess the consistency of the pairwise detection results among the three instruments, with the 95% consistency limit (mean ± 1.96 standard deviations) calculated as the consistency evaluation index. A polar coordinate scatter plot was used to illustrate the distribution of pupil shifts across the eyes. Statistical significance was set at *p* < 0.05.

## 3 Results

**Figure 1:**
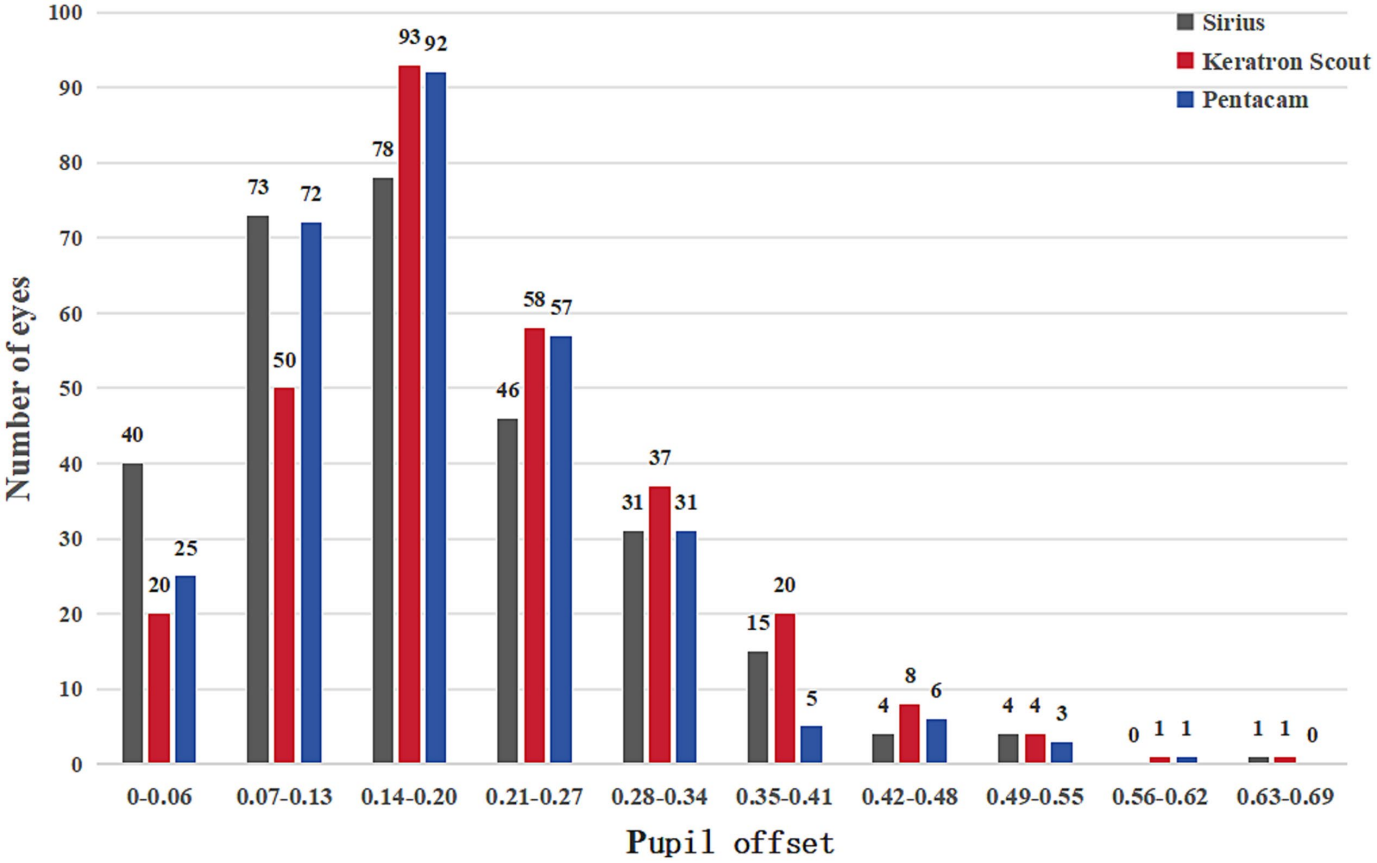
The Bland-Altman diagram illustrates the pairwise comparison of the consistency of the three devices in measuring the pupil offsets.

This study involved 292 eyes of 146 refractive candidates, comprising 81 males and 65 females. The demographic data and ocular parameters of the subjects are presented in Table 1. When measured by Pentacam, the average chord mu was 0.18 ± 0.10 (range: 0.02–0.57), the average Px was 0.00 ± 0.14 (range: −0.38 to 0.55), and the average Py was 0.09 ± 0.13 (range: −0.22 to 0.47). In contrast, when using the Keratron Scout to measure pupil offset, the average chord mu was 0.21 ± 0.11 (range: 0.01–0.64), the average Px was −0.02 ± 0.16 (range: −0.40 to 0.49), and the average Py was 0.10 ± 0.15 (range: −0.29 to 0.60). Measurements taken with Sirius indicated an average pupil offset chord length of 0.18 ± 0.11 (range: 0.01–0.69), an average Px of −0.01 ± 0.13 (range: −0.33 to 0.46), and an average Py of 0.10 ± 0.13 (range: −0.31 to 0.65). The overall difference in the repeated measurement variance analysis of pupil offset across different devices is statistically significant (*p* < 0.05; see Table 2). A histogram illustrating the chord-length distribution of pupil deviation, as measured by the three devices, is presented in Figure 1. The proportions of the Pentacam, Keratron Scout, and Sirius devices that recorded pupil offset chords mu greater than 0.20 mm were 36.3% (106 eyes), 44.2% (129 eyes), and 34.6% (101 eyes), respectively. Additionally, the proportions of chords mu exceeding 0.41 mm were 3.4% (10 eyes), 4.8% (14 eyes), and 3.1% (9 eyes), respectively.

**Table 1:** Demographic data and ocular parameters of the subjects.

Parameter	Mean ± SD	Range	95% CI
Number of eyes (n)	292		
Sex (male/female)	81/65		
Age, years	24.60 ± 7.01	17–52	23.79–25.41
Spherical, D	−4.59 ± 1.99	−10.25 to 0.25	−4.82 to 4.36
Cylindrical, D	−0.93 ± 0.68	−4.75 to 0	−1.00 to 0.85
BCVA	1.17 ± 0.08	0.8–1.2	1.16–1.18
Corneal keratometry, D	43.95 ± 1.60	37.4–49.1	43.76–44.13
Axial length, mm	26.03 ± 0.98	23.73–28.36	25.91–26.14

BCVA, best corrected visual acuity.

**Table 2:** Measurements of pupil offset and pupil diameter for three devices.

Parameter	Mean ± SD	Range	95% CI	F	*p* value
Chord Mu-Pentacam (mm)	0.18 ± 0.10	0.021–0.57	0.17–0.20	44.54	<0.001
Chord Mu-Keratron Scout (mm)	0.21 ± 0.11	0.01–0.64	0.20–0.22		
Chord Mu-Sirius (mm)	0.18 ± 0.11	0.01–0.69	0.17–0.19		
Px-Pentacam (mm)	0.00 ± 0.14	−0.38 to 0.55	−0.01 to 0.02	17.28	<0.001
Px-Keratron Scout (mm)	−0.02 ± 0.16	−0.40 to 0.49	−0.03 to 0		
Px-Sirius (mm)	−0.01 ± 0.13	−0.33 to 0.46	−0.02 to 0		
Py-Pentacam (mm)	0.09 ± 0.13	−0.22 to 0.47	0.07–0.10	4.50	0.012
Py-Keratron Scout (mm)	0.10 ± 0.15	−0.29 to 0.60	0.08–0.12		
Py-Sirius (mm)	0.10 ± 0.13	−0.31 to 0.65	0.08–0.11		
PD-Pentacam (mm)	3.26 ± 0.23	1.70–5.50	3.10–3.30	231.64	<0.001
PD-Keratron Scout (mm)	3.87 ± 0.55	2.25–5.77	3.77–3.94		
PD-Sirius (mm)	3.48 ± 0.50	2.30–5.27	3.37–3.55		

PD, pupil diameter; SD, standard deviation; CI, confidence interval.

Table 3 presents the results of the pairwise comparison of the repeated-measures ANOVA conducted on the pupil offset across different devices. There was no statistically significant difference between chord mu and Py of the Sirius and Pentacam, or between Px and Py of the Sirius and Keratron Scout, with *p*-values of 0.416, 0.237, 0.118, and 0.472, respectively. The differences in pupil offset indicators between the Keratron Scout and Pentacam were statistically significant (both *p* < 0.05).

**Table 3:** *Post hoc* test of repeated measures ANOVA on pupil offset measurements using three devices.

Parameter	Mean ± SD	95% CI	p value
Chord Mu: Sirius-Pentacam	−0.006	−0.015 to 0.004	0.416
Chord Mu: Sirius-Keratron Scout	−0.033	−0.041 to 0.025	<0.001
Chord Mu: Keratron Scout-Pentacam	0.027	0.018–0.037	<0.001
Px: Sirius-Pentacam	−0.014	−0.023 to 0.006	<0.001
Px: Sirius-Keratron Scout	0.007	−0.001 to 0.016	0.118
Px: Keratron Scout-Pentacam	−0.022	−0.032 to 0.012	<0.001
Py: Sirius-Pentacam	0.007	−0.003 to 0.018	0.237
Py: Sirius-Keratron Scout	−0.005	−0.014 to 0.004	0.472
Py: Keratron Scout-Pentacam	0.013	0.001–0.024	0.025
PD: Sirius-Pentacam	0.221	0.141–0.301	<0.001
PD: Sirius-Keratron Scout	−0.387	−0.448 to −0.327	<0.001
PD: Keratron Scout-Pentacam	0.609	0.544 to 0.674	<0.001

PD, pupil diameter; SD, standard deviation; CI, confidence interval.

**Figure 2:**
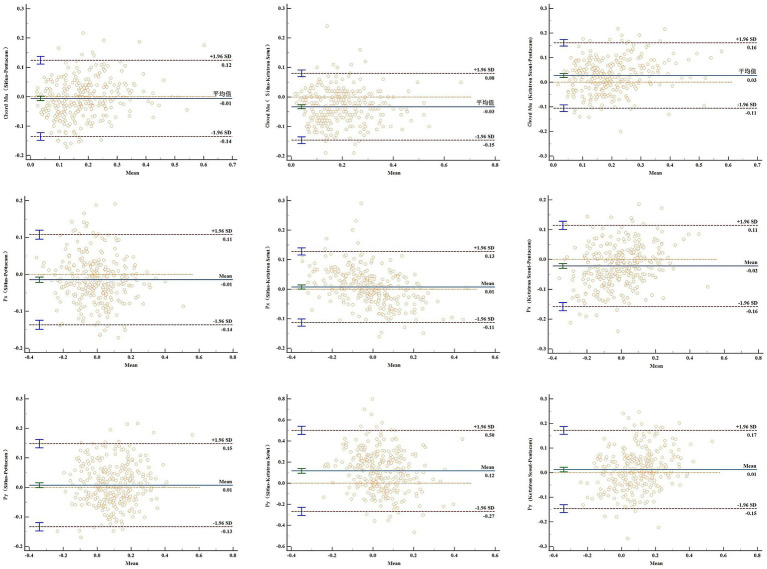
Histogram of pupil offset chord mu distribution measured using three devices.

As illustrated in Table 4, the chords mu, Px, and Py of the pupil deviation measured using the three devices exhibited good consistency with ICCs of 0.817 (0.783–0.847), 0.900 (0.880–0.917), and 0.8553 (0.8276–0.8797), respectively. As illustrated in Figure 2, the Bland–Altman method was employed to assess the consistency of the pupil offset and to calculate the 95% limits of agreement (LoA). The Sirius and Keratron Scout demonstrated strong consistency in measuring chord mu and Px, with the 95% LoA being the narrowest at −0.15 to 0.08 and − 0.11 to 0.13. Additionally, the measurement consistency of Py between Sirius and Pentacam was good, with the 95% LoA being the narrowest at −0.13 to 0.15.

**Table 4:** Intraclass correlation coefficient (ICC) test of pupil shift for three devices.

Parameter	ICC	95% CI
Chord Mu (mm)	0.817	0.783–0.847
Px (mm)	0.900	0.880–0.917
Py (mm)	0.855	0.828–0.880

ICC, intraclass correlation coefficient; CL, Confidence interval.

**Figure 3:**
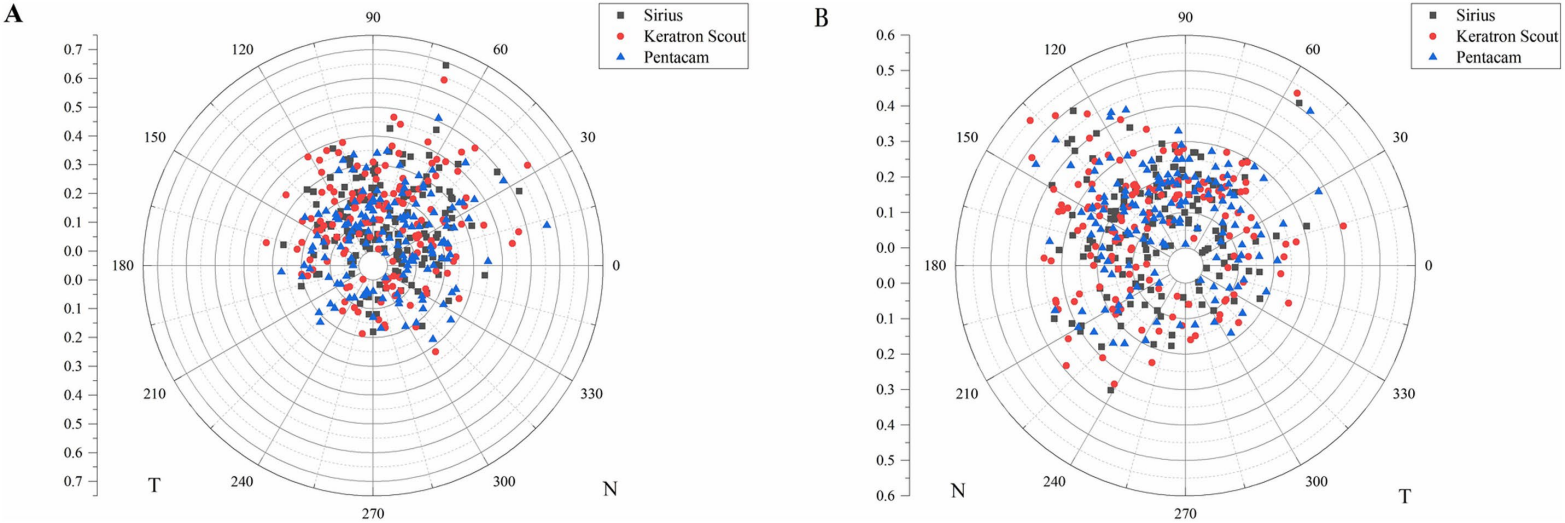
The distribution of pupil offset of the right (A) and left (B) eyes in the polar scatter plot. T: temporal, N: nasal.

Figure 3 illustrates the distribution of the pupil offsets. The upper half quadrants of both the left and right eyes exhibited a majority distribution. Specifically, the distribution percentages of the Pentacam, Keratron Scout, and Sirius devices in the upper half quadrant of the right eye were 71.92% (105 eyes), 77.40% (113 eyes), and 82.88% (121 eyes), respectively. The corresponding percentages for the left eye were 79.45% (116 eyes), 71.92% (105 eyes), and 74.66% (109 eyes).

## 4 Discussion

The cautery center for corneal ablation surgery is crucial for achieving optimal visual quality during the postoperative period. Most laser corneal refractive surgery platforms use the pupil center as a reference point. However, in cases with a large kappa angle, using the pupil as the cutting center can result in eccentric cutting, which may increase high-order aberrations, glare, and halos following surgery as well as visual quality issues such as monocular diplopia and diminished night vision (5, 10, 11). Reinstein et al. revealed that utilizing the corneal apex as the ablation center strategy resulted in no significant differences in postoperative safety, accuracy, induced astigmatism, contrast sensitivity, or night vision impairment between the two groups with pupil offsets chord mu of <0.25 mm and > 0.55 mm (12). The corneal vertex, being closer to the ideal cutting center (visual axis), serves as a more stable and preferred reference center for surgical procedures (2, 6). Accurate measurement of the relative position of the pupil center and the corneal apex (pupil offset) is critical to the success of surgery. Corneal topography devices such as Pentacam, Keratron Scout, and Sirius are commonly used to obtain pupil offset measurements. However, the measurement principles of these three instruments differ, and there are currently relatively few comparative studies that assess pupil deflection using all three instruments simultaneously. Therefore, this study aimed to compare the consistency and differences in pupil deflection measurement results among the three instruments to provide insights for clinical applications.

Previous research has demonstrated that the intra-class correlation coefficients of the Pentacam and Keratron Scout for measuring the pupil offset chords mu, Px, and Py in young myopic individuals are 0.82, 0.84, and 0.81, respectively (13). These findings suggest that the two devices exhibited good consistency. Furthermore, this study demonstrates that the intraclass correlation coefficients (ICC) for the three devices measuring pupil deviation, chord mu, Px, and Py, were 0.817, 0.900, and 0.855, respectively. These values are consistent with or even superior to those reported in the aforementioned study, indicating that the three devices exhibit strong consistency. The 95% consistency limit analysis of pupil offset indicated that Sirius and Keratron Scout exhibited superior consistency in measuring chord mu and Px, whereas Sirius and Pentacam demonstrated enhanced consistency in measuring Py. In clinical practice, it is recommended that more consistent equipment be used to compare different pupil offset components to enhance the accuracy of data collection.

This study found that the pupil offset chord mu, as measured using Pentacam, Keratron Scout, and Sirius, were 0.18 ± 0.10, 0.21 ± 0.11, and 0.18 ± 0.11, respectively. Sun et al. used Pentacam to assess pupil offset characteristics in Asians with high myopia (14). The average chord mu was found to be 0.18 ± 0.09 mm, which aligns closely with our findings, suggesting that young individuals in the same region exhibit similar pupil offset values. Reinstein et al. used Orbscan II to assess the pupil offset in 125 individuals with 250 myopic eyes (15). They reported a chord mu of 0.27 ± 0.14, which is greater than the measurements obtained in our study. The observed differences may be attributed to variations in the measuring equipment, as well as those related to race and region. Previous studies have demonstrated that, in wavefront aberration-guided corneal refractive surgery, a decentration of <0.2 mm can preserve good optical quality when the pupil diameter is 3.0 mm (16). Liu et al. analyzed the relationship between postoperative higher-order aberrations and preoperative pupil offset following femtosecond laser-assisted *in situ* keratomileusis (5). They found that when the chord mu exceeded 0.2 mm, there was a more pronounced increase in postoperative higher-order aberrations. In this study, the proportion of pupil offset was greater than 0.2 mm, while the chord mu ranged from 34.6 to 44.3%. Additionally, the proportion of chord mu exceeding 0.4 mm was between 3.1 and 4.8%, which was consistent with the findings of Sun et al. (14). Therefore, individuals with significant pupil deviation should prioritize the alignment strategy of the cutting center and adjust the pupil offset to ensure optimal postoperative visual quality.

Through repeated-measures analysis of variance, post-hoc pairwise comparisons revealed that the differences in string mu, Px, and Py between the Pentacam and Keratron Scout, as well as the differences in Px between the Pentacam and Sirius and in string mu between the Keratron Scout and Sirius, were all statistically significant. The differences in certain pupil offset parameters between the three devices can be attributed to several factors. The measurement principles were different for three devices. Pentacam employs a 360-degree rotating Scheimpflug camera to conduct tomographic scanning of the cornea. It captures 25 cross-sectional Scheimpflug images within a span of 2 s and gathers 69,000 data points to reconstruct the three-dimensional structure of the cornea (17, 18). The Keratron Scout corneal topographer measures the shape of the anterior surface of the cornea using a small cone Placido disk, providing instantaneous measurement (8). Siriu integrated a rotating Scheimpflug camera with a corneal topography system and a Placido disk, enabling the acquisition of 35,632 points on the anterior surface of the cornea and 30,000 points on the posterior surface. This combination produced reliable anterior segment measurements (7).

The distribution of the pupil center relative to the corneal apex can be represented by the XY Cartesian coordinate system. Qin et al. analyzed data from 113 patients with cataracts, comprising 60 right eyes and 53 left eyes (19). Their study revealed that the pupil centers of most patients were located on the temporal side of the corneal apex. In contrast, our study indicated that the pupil centers of both eyes were predominantly distributed in an upward direction. These differences may be attributed to variations in age distribution, lens transparency, and sample sizes between the two studies. Differences in the pupil diameter measurements obtained using various devices may result in different pupil offsets. Although all three devices were utilized in the same examination room during the measurement process, discrepancies in color, intensity, and range of illumination across different devices can lead to variations in pupil diameter. Our results indicate that the pupil diameter, ranked from largest to smallest, is as follows: Keratron Scout, Sirius, and Pentacam.

Huang et al. found that in eyes with high astigmatism undergoing femtosecond laser small incision microlens extraction, an eccentricity >0.2 mm results in increased coma and spherical aberration following surgery (20). Liu et al. found that for individuals undergoing SMILE surgery when the chord mu was <0.2 mm, there was no significant difference in postoperative total eccentric displacement and higher-order aberration between the pupil center group and the tear film mark center group (21). The average differences in the string mu of the three devices range from 0.01 to 0.03 mm, with Px measuring between 0.01 and 0.02 mm and Py at 0.01 mm. Although these differences were statistically significant, they did not have a significant impact on clinical practice.

This study had several limitations. First, the population primarily included myopic individuals planning to undergo refractive surgery, whereas emmetropic and hyperopic individuals were not represented. Research has indicated that there are notable differences in pupil deviation among individuals with varying refractive statuses (15). Second, pupillary shifts have been extensively utilized in the field of refractive cataract surgery (19). The population included in this study predominantly comprised younger individuals; older adults were excluded. Finally, this study did not comprehensively account for other ocular parameters. Future research should aim to incorporate a larger sample size, a broader spectrum of refractive statuses, and age distributions to analyze the relationship between additional ocular parameters and pupil deviation, thereby providing valuable insights into the clinical practice of refractive surgery.

## 5 Conclusion

In summary, the Pentacam, Keratron Scout, and Sirius exhibited strong consistency in measuring pupillary shifts among young patients with myopia. Consequently, the three devices can be used as reliable references for one another in clinical practice.

## Data Availability

The raw data supporting the conclusions of this article will be made available by the authors, without undue reservation.

## References

[1] Chung B Lee H Roberts CJ Kang DSY Reinstein DZ Jean SK. Decentration measurements using Placido corneal tangential curvature topography and Scheimpflug tomography pachymetry difference maps after small-incision lenticule extraction. J Cataract Refract Surg. (2019) 45:1067–73. doi: 10.1016/j.jcrs.2019.03.019, PMID: 31133417

[2] Mosquera SA Verma S McAlinden C. Centration axis in refractive surgery. Eye Vis. (2015) 2:4. doi: 10.1186/s40662-015-0014-6, PMID: 26605360 PMC4655455

[3] Lee H Roberts CJ Arba-Mosquera S Kang DSY Reinstein DZ Kim TI. Relationship between decentration and induced corneal higher-order aberrations following small-incision lenticule extraction procedure. Invest Ophthalmol Vis Sci. (2018) 59:2316–24. doi: 10.1167/iovs.17-23451, PMID: 29847636

[4] Lee SB Hwang BS Lee J. Effects of decentration of photorefractive keratectomy on the induction of higher order wavefront aberrations. J Refract Surg. (2010) 26:731–43. doi: 10.3928/1081597X-20091209-01, PMID: 20027991

[5] Liu Z Zhao Y Sun S Wu Y Wang G Zhao S. Effect of preoperative pupil offset on corneal higher-order aberrations after femtosecond laser-assisted in situ keratomileusis. BMC Ophthalmol. (2023) 23:247. doi: 10.1186/s12886-023-02960-y, PMID: 37264322 PMC10236578

[6] Zhang J Wang Y Chen X Wu W. Clinical outcomes of corneal refractive surgery comparing centration on the corneal vertex with the pupil center: a meta-analysis. Int Ophthalmol. (2020) 40:3555–63. doi: 10.1007/s10792-020-01506-1, PMID: 32671600

[7] Savini G Barboni P Carbonelli M Hoffer KJ. Repeatability of automatic measurements by a new Scheimpflug camera combined with Placido topography. J Cataract Refract Surg. (2011) 37:1809–16. doi: 10.1016/j.jcrs.2011.04.033, PMID: 21852068

[8] Fityo S Bühren J Shajari M Kohnen T. Keratometry versus total corneal refractive power: analysis of measurement repeatability with 5 different devices in normal eyes with low astigmatism. J Cataract Refract Surg. (2016) 42:569–76. doi: 10.1016/j.jcrs.2015.11.046 27113880

[9] Koo TK Li MY. A guideline of selecting and reporting intraclass correlation coefficients for reliability research. J Chiropr Med. (2016) 15:155–63. doi: 10.1016/j.jcm.2016.02.012, PMID: 27330520 PMC4913118

[10] Park CY Oh SY Chuck RS. Measurement of angle kappa and centration in refractive surgery. Curr Opin Ophthalmol. (2012) 23:269–75. doi: 10.1097/ICU.0b013e3283543c41, PMID: 22569467

[11] Liu M Sun Y Wang D Zhang T Zhou Y Zheng H. Decentration of optical zone center and its impact on visual outcomes following SMILE. Cornea. (2015) 34:392–7. doi: 10.1097/ICO.0000000000000383, PMID: 25742387

[12] Reinstein DZ Gobbe M Archer TJ. Coaxially sighted corneal light reflex versus entrance pupil center centration of moderate to high hyperopic corneal ablations in eyes with small and large angle kappa. J Refract Surg. (2013) 29:518–25. doi: 10.3928/1081597X-20130719-08, PMID: 23909778

[13] Sun S Sun M Tang J Yang F Liu Z Zhao S. A comparative study of pupil offset measurement using Pentacam and Keratron scout in myopic young adults. Clin Exp Optom. (2024) 107:40–6. doi: 10.1080/08164622.2023.2203316, PMID: 37156100

[14] Sun S Liu Z Wu Y Sun X Zhao S Huang Y. Characteristics of pupil offset in young asian adults with mild-moderate and high myopia. Transl Vis Sci Technol. (2022) 11:13. doi: 10.1167/tvst.11.6.13, PMID: 35696132 PMC9202332

[15] Reinstein DZ Archer TJ Rowe EL Gobbe M Vida RS. Distribution of pupil offset and angle kappa in a refractive surgery preoperative population of 750 myopic, emmetropic, and hyperopic eyes. J Refract Surg. (2021) 37:49–58. doi: 10.3928/1081597X-20201109-01, PMID: 33432995

[16] Bueeler M Mrochen M Seiler T. Maximum permissible lateral decentration in aberration-sensing and wavefront-guided corneal ablation. J Cataract Refract Surg. (2003) 29:257–63. doi: 10.1016/S0886-3350(02)01638-3, PMID: 12648634

[17] Kanellopoulos AJ. Scheimpflug vs scanning-slit corneal tomography: comparison of corneal and anterior chamber tomography indices for repeatability and agreement in healthy eyes. Clin Ophthalmol. (2020) 14:2583–92. doi: 10.2147/OPTH.S251998, PMID: 32943840 PMC7481306

[18] Herber R Lenk J Pillunat LE Raiskup F. Agreement and repeatability of corneal tomography in healthy eyes using a new swept-source OCT, a rotating Scheimpflug camera, and a dual Scheimpflug-Placido system. J Cataract Refract Surg. (2022) 48:190–8. doi: 10.1097/j.jcrs.0000000000000734, PMID: 34224476

[19] Qin M Yuan Y Wang Y Li P Chen W Wang Y. Comparison of preoperative angle kappa measurements in the eyes of cataract patients obtained from Pentacam Scheimpflug system, optical low-coherence reflectometry, and ray-tracing aberrometry. BMC Ophthalmol. (2022) 22:153. doi: 10.1186/s12886-021-02116-w, PMID: 35366842 PMC8976989

[20] Huang J Zhou X Qian Y. Decentration following femtosecond laser small incision lenticule extraction (SMILE) in eyes with high astigmatism and its impact on visual quality. BMC Ophthalmol. (2019) 19:151. doi: 10.1186/s12886-019-1153-7, PMID: 31315595 PMC6637638

[21] Liu S Zhang X You Z Zhou X. Comparison of the distribution of lenticule decentration following SMILE by pupil center or tear film mark centration. J Refract Surg. (2020) 36:239–46. doi: 10.3928/1081597X-20200310-01, PMID: 32267954

